# Supplementary figures and images for: Using medical claims database to develop a population disease progression model for leuprorelin-treated subjects with hormone-sensitive prostate cancer
Source: PLoS One. 2020 Mar 24;15(3):e0230571. doi: 10.1371/journal.pone.0230571 (PMC7092991; doi:10.1371/journal.pone.0230571)

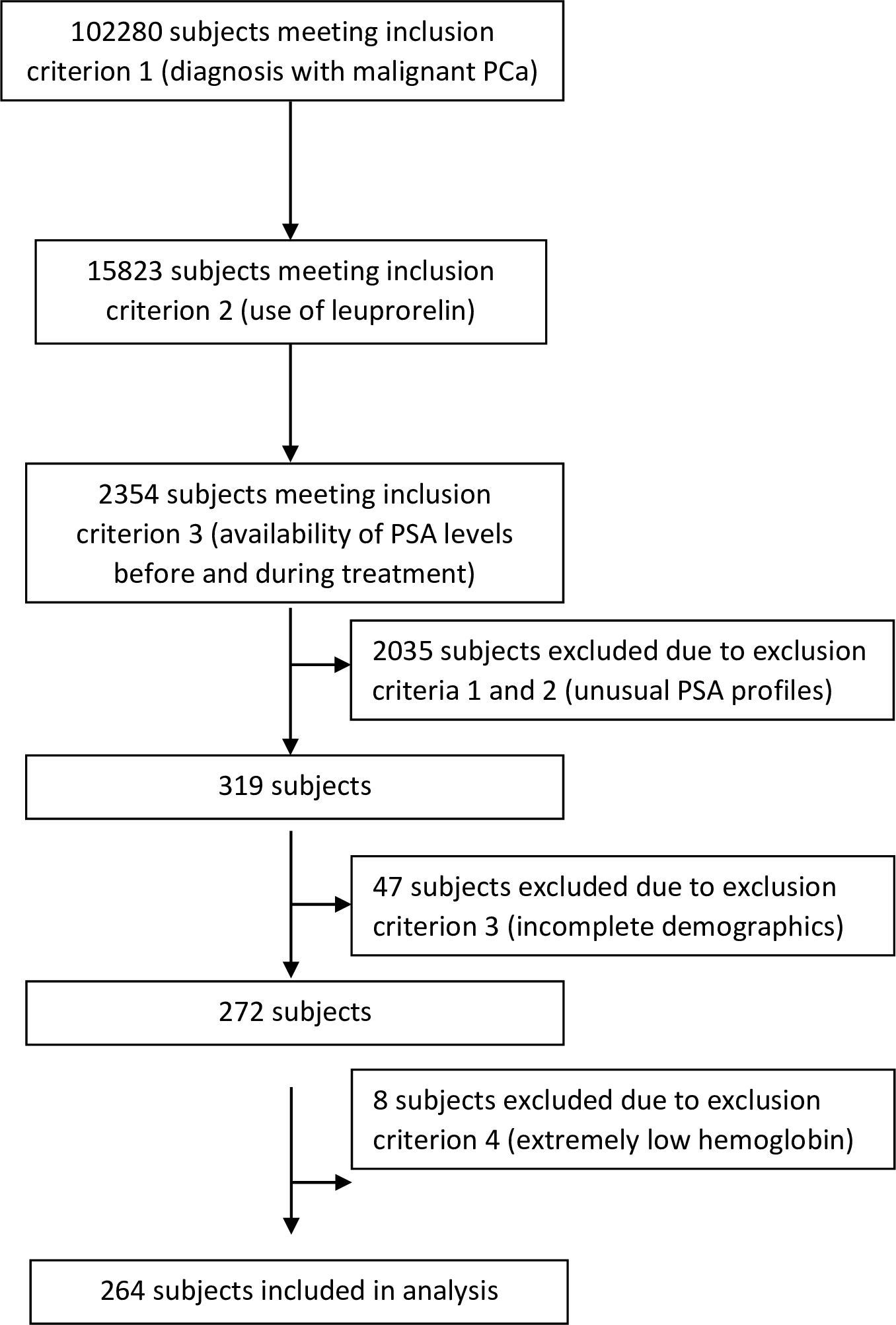

Supplement: S1 Fig — (TIF) [file pone.0230571.s001.tif]

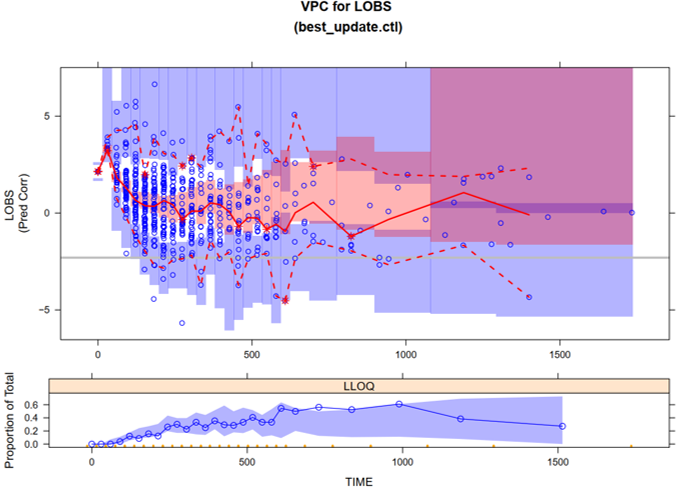

Supplement: S2 Fig — Blue circles represent prediction-corrected observations (log-transformed PSA concentrations). The solid red line represents the median prediction-corrected observations, and the semitransparent red area represents the simulated 95% confidence interval for the median. The dashed red lines represent the 5% and 95% percentiles of prediction-corrected observations, and the semitransparent blue areas represent their respective simulated 95% confidence intervals. (TIF) [file pone.0230571.s002.tif]
